# Preserved vegetable consumption and gastrointestinal tract cancers: A prospective study

**DOI:** 10.7189/jogh.14.04191

**Published:** 2024-11-08

**Authors:** Wei Yu, Yalei Ke, Jun Lv, Dianjianyi Sun, Pei Pei, Ling Yang, Yiping Chen, Huaidong Du, Kaixu Xie, Xiaoming Yang, Maxim Barnard, Junshi Chen, Zhengming Chen, Liming Li, Canqing Yu, The China Kadoorie Biobank Collaborative Group

**Affiliations:** 1 Department of Epidemiology and Biostatistics, School of Public Health, Peking University, Beijing, China; 2 Centre for Public Health and Epidemic Preparedness and Response, Peking University, Beijing, China; 3 Key Laboratory of Epidemiology of Major Diseases, Peking University, Ministry of Education, Beijing, China; 4 State Key Laboratory of Vascular Homeostasis and Remodelling, Peking University, Beijing, China; 5 Clinical Trial Service Unit and Epidemiological Studies Unit, Nuffield Department of Population Health, University of Oxford, Oxford, UK; 6 Noncommunicable Diseases Prevention and Control Department, Tongxiang Centre for Disease Control and Prevention, Jiaxing, China; 7 China National Centre for Food Safety Risk Assessment, Beijing, China

## Abstract

**Background:**

This study aimed to assess the associations of two common types of preserved vegetables in China, salted and sour pickled vegetables, with the risk of gastrointestinal tract (GI) cancers, including oesophageal cancer, stomach cancer, and colorectal cancer.

**Methods:**

The China Kadoorie Biobank collected intake frequency of preserved vegetables among 510 143 adults without self-reported cancer during 2004–2008, and followed up till 31 December 2018. The second resurvey further collected intake frequencies of salted and sour pickled vegetables, which classified the 10 study areas into three types of regions, including the regions never/rarely consuming preserved vegetables (number of participants at baseline = 201 844), mainly consuming salted vegetables (n = 202 927), and mainly consuming sour pickled vegetables (n = 105 372). Cox proportional models were respectively performed to calculate hazard ratios (HRs) for GI cancers with preserved vegetables in the latter two types of regions among baseline participants.

**Results:**

In the regions mainly consuming salted vegetables, preserved vegetable consumption was positively associated with stomach cancer (HR = 1.17; 95% confidence interval = 1.00–1.37; *P* for trend = 0.039). In the regions mainly consuming sour pickled vegetables, a dose-response positive relationship was observed between preserved vegetable consumption and the risk of oesophageal cancer (*P* for trend = 0.013), with adjusted HR of 1.35 (95% CI 1.02–1.80) for those who daily consumed compared with never consumed.

**Conclusions:**

Our findings suggest that different types of preserved vegetables might have different effects on GI cancers, and limiting preserved vegetable consumption might be protective against developing GI cancers.

Worldwide, gastrointestinal tract (GI) cancers accounted for approximately one-fifth of the incidence and mortality of all cancers, with 3.62 million newly-diagnosed cases and 2.25 million deaths in 2020 [1]. GI cancers pose a severe disease burden in China and exhibit unique characteristics. The National Cancer Centre of China reported 1.16 million cases and 0.68 million deaths due to GI cancers in 2016 [2]. Oesophageal and stomach cancer incidence and mortality rates in China are significantly higher than those in Western countries such as the United Kingdom and the United States [1]. Moreover, squamous cell carcinoma accounts for over 90% of oesophageal cancer in China, whereas adenocarcinoma is the predominant subtype in Western countries [3]. Therefore, it is imperative to research the risk factors of GI cancers in China.

Diet is a modifiable risk factor for GI cancers. Preserved vegetables offer the advantages of long preservation and unique flavours, which has led to their popularity worldwide. Well-known preserved vegetables include American sour cucumber, German red cabbage, Korean kimchi, Japanese tsukemono, and pickles in the Sichuan (a province in China) style. Preserved vegetables are distinguished by tastes and processing methods and could be typically classified into salted vegetables and sour pickled vegetables accordingly. Salted vegetables are made by permeating in high-concentration salt, such as Meicai, a kind of salted-and-sun-dried Chinese cabbage. Sour pickled vegetables are made from fermentation by lactic acid bacteria and other microorganisms, such as pickled vegetables in the Sichuan style and Chinese sauerkraut in the Northeastern style.

The association between preserved vegetables and the risk of GI cancers has not been fully clarified. A recent meta-analysis of 10 cohort studies (eight among Japanese and two among Korean) revealed a positively dose-response relationship between preserved vegetable consumption and the risk of stomach cancer [4]. However, such association was only found among Japanese, but not among Korean [4]. For Chinese, a positive association between preserved vegetable consumption and the risk of stomach cancer was found in a meta-analysis of 27 case-control studies and two cohort studies [5]. The regional disparity might arise from the distinct processing methods of preserved vegetables, resulting in the variations in types [6,7]. Therefore, it is essential to fully consider the varied types of preserved vegetables across different regions. As for oesophageal cancer, previous case-control studies discovered a positive association between preserved vegetables and oesophageal cancer [8]. However, cohort studies conducted in Linxian, a high-risk area for oesophageal cancer in China, did not find a pronounced association, which might be attributed to implementing anti-cancer public health campaigns that proposed to limit the intake of preserved vegetables [9,10]. Until now, limited research examined the association between preserved vegetables and colorectal cancer, which is one of the most common cancers in China and worldwide in 2020 [1].

To address the evidence gap, we conducted the present study to explore the association of two major types of preserved vegetable consumption, i.e. salted and sour pickled vegetables, with the incidence of three major GI cancers (i.e. oesophageal, stomach, and colorectal cancers) using China Kadoorie Biobank (CKB) study.

## METHODS

### Study design and participants

The CKB study is a population-based prospective cohort study. At the baseline survey in 2004–2008, 512 723 individuals aged 30–79 years from five urban areas (Liuzhou, Haikou, Suzhou, Qingdao, and Harbin) and five rural areas (Sichuan, Gansu, Hunan, Henan, and Zhejiang) across China participated in a questionnaire survey, anthropometric measurements, and blood draw. Besides, periodic resurveys were conducted every 4–5 years among ~ 5% randomly selected participants. Using similar but more detailed procedures, resurveys are good supplements to the baseline survey. Details of the CKB study have been described elsewhere [11,12].

In the present study, we excluded the participants with a self-reported history of cancer (n = 2 578), and those with missing values of body mass index (BMI) (n = 2), leaving 510 143 in the present analysis (Figure S1 in the **Online Supplementary Document**).

### Assessment of preserved vegetable consumption

An interviewer-administered electronic questionnaire was employed to collect information on dietary habits (including preserved vegetable consumption) and other covariates, namely sociodemographic characteristics (age, sex, study regions, highest educational level, and household income), smoking status, alcohol intake, physical activity, et al.

Specifically, dietary habits were assessed through a validated self-designed food frequency questionnaire (FFQ) at baseline [13]. Participants were asked about intake frequency (never/rarely, monthly, 1–3 days/week, 4–6 days/week, or daily) of each food item last year. Items of the FFQ covered major common food groups in China, including rice, wheat products, other staple foods, meat, poultry, fish, eggs, dairy products, soybean products, fresh fruit, fresh vegetables, and preserved vegetables (such as Meicai and pickled mustard).

At the second resurvey in 2013–2014, preserved vegetables were divided up into salted vegetables and sour pickled vegetables, and were respectively collected the intake frequency and amount. The more detailed data of the resurvey made it possible to estimate the habitual intake type and usual amount of preserved vegetables at baseline, which enabled us to examine the association of different types of preserved vegetables with health outcomes and correct regression dilution bias (Supplementary methods in the **Online Supplementary Document**).

Two dietary validation studies of 432 and 416 participants confirmed acceptable or good relative validity compared to 12-day 24-hour dietary recalls and reproducibility of the qualitative FFQ at baseline and quantitative FFQ at the second resurvey. About preserved vegetables of the qualitative FFQ, the weighted kappa coefficients for the validity and reproducibility were 0.81 and 0.75, respectively. Similarly, the salted vegetables and sour pickled vegetables of the quantitative FFQ also showed good relative validity and reproducibility, with weighted kappa coefficients >0.80 (for salted vegetables) and adjusted spearman coefficients ~ 0.70 (for sour pickled vegetables) [13].

### Identification of cancer cases

The CKB study followed up with participants through multiple methods, such as local disease and death registries, electronic linkage to the national health insurance system, and annual active follow-up, which ensured a low percentage (<1%) of loss to follow-up. The ongoing event adjudication has checked the accuracy of incident cancer in the study. By 31 December 2017, professional doctors reviewed the medical notes of 16 998 cancer cases, and ~ 95% of primary diagnoses were confirmed. Trained staff blinded to the baseline information coded the non-fatal and fatal events by the International Classification of Diseases (ICD)-10. The present study focused on GI cancers, including oesophageal cancer (ICD-10: C15), stomach cancer (C16), and colorectal cancer (C18–C20). The person-years were calculated from enrolment to the date of the outcome of interest, death, loss to follow-up, or the study end date (31 December 2018 for this present study), whichever came first.

### Statistical analyses

The present study classified the 10 survey areas according to the type of preserved vegetables mainly consumed at the second resurvey (Supplementary methods, Figure S2, Figure S3 in the **Online Supplementary Document**). Three types of regions were classified:

1) rarely consuming both salted vegetables and sour pickled vegetables, abbreviated as ‘never/rarely consuming preserved vegetables’ (Haikou, Liuzhou, Hunan, and Henan, number of participants at baseline = 201 844)

2) often consuming salted vegetables but rarely consuming sour pickled vegetables, abbreviated as ‘mainly consuming salted vegetables’ (Qingdao, Harbin, Suzhou, and Zhejiang, n = 202 927)

3) often consuming sour pickled vegetables but rarely consuming salted vegetables, abbreviated as ‘mainly consuming sour pickled vegetables’ (Sichuan and Gansu, n = 105 372). Considering eating habits were a long-established tradition and culture, we assumed that in each study area, the average intake frequency of each type of preserved vegetables would not change drastically over time. Therefore, the following analyses were performed in the regions mainly consuming salted vegetables and the regions mainly consuming sour pickled vegetables.

The histograms were drawn to check whether the continuous variables followed the normal distribution. The baseline characteristics of participants across the five categories of baseline preserved vegetable consumption were described as mean (standard deviations (SDs)) or median (interquartile range (IQR)) for continuous variables and percentages for categorical variables. Cox proportional regression models were performed to calculate hazard ratios (HRs) and 95% confidence intervals (CIs) for GI cancers with preserved vegetable consumption, with participants who never/rarely consumed preserved vegetables (defined as ‘rare consumers’ hereafter) as the reference group. Using age as underlying time scale, the Cox models were stratified by baseline age groups (in five-year intervals) and adjusted potential confounders including education (no formal school, primary school, middle school, high school, college and above), household income (<2500, 2500–4999, 5000–9999, 10 000–19 999, 20 000–34 999, ≥35 000 yuan/y), family history of cancer (yes/no), smoking (never/occasional smokers, former smokers, current 1–9 cigarettes/d, 10–19 cigarettes/d, 20–29 cigarettes/d, ≥30 cigarettes/d), alcohol consumption (not weekly drinking, ex-regular drinkers, weekly drinkers, daily and <15 g/d, 15–29 g/d, 30–59 g/d, ≥60 g/d) [14], physical activity (continuous, metabolic equivalent of task hours per day (MET-h)), BMI (continuous, kg/m^2^), waist circumference (continuous, cm), and intake frequency of fresh fruits, meat (never/rarely, monthly, 1–3 days/week, 4–6 days/week, daily), fresh vegetables (daily or not daily), and spicy food (never/occasionally, monthly, 1–2 days/week, 3–5 days/week, daily/almost every day). The proportional hazards assumptions of Cox models were not violated via Schoenfeld residuals. We took the intake frequency of preserved vegetables as a continuous variable to estimate the dose-response relationship. Besides, we corrected regression dilution bias by estimating the usual amount of preserved vegetables (Supplementary methods in the **Online Supplementary Document**).

Several sensitivity analyses were conducted to assess the robustness of the main results. First, we excluded the participants who developed corresponding cancer during the first two years to eliminate the reverse causation in analysing each site-specific GI cancer. Second, we additionally adjusted a few variables to reduce the possibility of residual confounding. Specifically, we respectively further adjusted:

1) daily energy intake (continuous in log-transformed formal, kcal/d)

2) intake of dietary supplements (yes or no), including fish oil/cod liver oil, vitamins, calcium/iron/zinc, ginseng, and other herbal products

3) the years of having a refrigerator (continuous, years), an indicator for the food preservation

4) intake of rice, wheat, other staples, poultry, fish/seafood, eggs, soybean, and dairy products (never/rarely, monthly, 1–3 days/week, 4–6 days/week, daily)

5) the frequency and temperature of tea drinking (less than weekly, weekly, daily and warm, daily and hot, daily and burning hot).

We also examined whether the association of preserved vegetables and GI cancers varied in different subgroups stratified by age, sex, smoking status (only in males due to the scarcity of current female smokers), alcohol drinking status (only in males), body shape (defined by BMI and waist circumference), the temperature of tea drinking, and the intake frequency of spicy food, fresh fruit, and meat. The likelihood ratio tests calculated the *P*-values of interaction.

All statistical analyses were conducted by Stata (version 15.0, Texas, USA, copyright 1985-2024). All *P-*values were two-sided, and statistical significance was defined as *P* < 0.05.

## RESULTS

Salted vegetable-consuming regions were three urban areas (Qingdao, Harbin, and Suzhou) and one ‘semi-rural’ area (Zhejiang). Among the participants in these regions (n = 202 927, median age: 51.6 (IQR = 16.5) years, 58.2% were females), 29.5% consumed daily with an average consumption of 3.1 days/week. Whereas sour pickled vegetable-consuming regions were two rural areas (Sichuan and Gansu, n = 105 372, median age: 50.4 (17.0) years, and 61.5% were females), and had an average intake of 2.8 days/week, with 16.3% consumed daily. Overall, participants from salted vegetable-consuming regions had a higher education level, household income, and a longer time owning a refrigerator than those in sour pickled vegetable-consuming regions (**Table 1**, **Table 2**, Table S1, Table S2 in the **Online Supplementary Document**).

**Table 1:** Baseline characteristics of participants in the regions mainly consuming salted vegetables (n = 202 927)*

Characteristics	Frequency of preserved vegetable consumption					
	**Never**	**Monthly**	**1–3 day/week**	**4–6 day/week**	**Daily**	**Overall**
Number of participants	25 416	47 169	54 489	15 926	59 927	202 927
Usual preserved vegetable consumption (g/d)	4.5 (2.1)	7.5 (1.6)	13.9 (2.2)	20.7 (3.8)	29.3 (9.4)	13.9 (18.4)
Age (years)	51.5 (17.9)	51.5 (16.1)	50.8 (16.3)	51.4 (15.8)	52.6 (16.2)	51.6 (16.5)
Females (%)	59.9	58.2	56.5	58.1	59.0	58.2
Education ≥ middle school (%)	74.5	43.2	50.9	50.3	54.2	53.0
Household income ≥20 000 yuan/y (%)	60.8	73.5	71.1	67.2	61.2	67.2
Family history of cancer (%)	22.7	17.0	18.8	21.0	24.4	20.7
Ever smokers in males (%)	78.4	87.1	85.2	86.4	88.0	85.8
Ever smokers in females (%)	5.7	3.7	3.9	4.7	5.9	4.7
Weekly alcohol consumption in males (%)	39.9	39.1	42.5	44.9	50.3	43.8
Weekly alcohol consumption in females (%)	2.7	1.8	1.9	2.2	2.3	2.1
Regular consumption of foods (%)						
*Fresh vegetable (daily)*	97.8	96.2	96.3	97.1	98.8	97.3
*Fresh fruit (>four days/week)*	58.0	30.7	37.7	41.8	41.8	40.1
*Meat (>four days/week)*	58.9	53.1	55.4	55.8	54.0	54.9
*Spicy food (daily)*	4.8	3.7	4.4	5.0	9.2	5.8
Total physical activity (MET-h/d)	15.0 (17.6)	22.9 (24.6)	21.8 (23.4)	20.9 (23.0)	17.6 (21.3)	19.6 (22.4)
BMI (kg/m^2^)	24.6 (3.5)	23.6 (3.4)	24.0 (3.4)	24.2 (3.4)	24.6 (3.4)	24.2 (3.4)
Waist circumference (cm)	82.5 (10.3)	79.3 (9.9)	80.3 (10.1)	80.9 (10.0)	82.3 (10.1)	81.0 (10.1)
Years of owning a refrigerator (years)	15.0 (11.0)	5.0 (14.0)	8.0 (15.0)	8.0 (15.0)	10.0 (13.0)	10.0 (15.0)

MET-h/d – metabolic equivalent task hours per day, BMI – body mass index

*The regions mainly consuming salted vegetables included Qingdao, Harbin, Suzhou, and Zhejiang. For continuous variables, BMI and waist circumference were described as mean (standard deviation), and others were described as median (interquartile range). Categorical variables were described as percentages.

**Table 2:** Baseline characteristics of participants in the regions mainly consuming sour pickled vegetables (n = 105 372)*

Characteristics	Frequency of preserved vegetable consumption					
	**Never**	**Monthly**	**1–3 day/week**	**4–6 day/week**	**Daily**	**Overall**
Number of participants	10 621	16 575	44 886	16 134	17 156	105 372
Usual preserved vegetable consumption (g/d)	3.9 (1.3)	7.5 (4.0)	15.0 (5.1)	23.0 (2.6)	29.5 (5.5)	15.0 (12.6)
Age (years)	53.7 (18.9)	49.8 (16.9)	49.4 (16.8)	50.8 (17.0)	50.6 (16.2)	50.4 (17.0)
Females (%)	62.4	62.0	61.3	63.8	59.1	61.5
Education ≥ middle school (%)	30.0	33.3	30.6	25.6	35.7	31.0
Household income ≥20 000 yuan/y (%)	7.5	6.2	4.5	4.3	9.1	5.8
Family history of cancer (%)	11.3	10.8	10.2	11.1	14.3	11.2
Ever smokers in males (%)	87.0	87.4	87.1	88.7	90.0	87.9
Ever smokers in females (%)	21.4	11.1	7.7	8.6	18.1	11.4
Weekly alcohol consumption in males (%)	31.9	25.6	23.3	23.2	55.7	30.1
Weekly alcohol consumption in females (%)	4.3	2.5	2.2	2.4	7.7	3.4
Regular consumption of foods (%)						
*Fresh vegetable (daily)*	96.1	86.0	82.3	78.3	97.1	86.1
*Fresh fruit (>four days/week)*	21.2	16.5	16.6	19.6	27.9	19.3
*Meat (>four days/week)*	37.2	27.4	24.4	25.5	49.8	30.5
*Spicy food (daily)*	53.9	54.7	54.0	55.5	74.4	57.6
Total physical activity (MET-h/d)	20.2 (16.1)	23.8 (16.0)	26.1 (16.3)	26.3 (16.9)	20.8 (16.0)	24.4 (16.8)
BMI (kg/m^2^)	22.9 (3.2)	23.0 (3.2)	23.0 (3.2)	23.0 (3.2)	23.3 (3.2)	23.0 (3.2)
Waist circumference (cm)	77.7 (9.2)	78.7 (9.2)	79.3 (9.3)	79.3 (9.2)	78.5 (9.1)	78.9 (9.2)
Years of owning a refrigerator (years)	0.0 (0.0)	0.0 (0.0)	0.0 (0.0)	0.0 (0.0)	0.0 (1.0)	0.0 (0.0)

MET-h/d – metabolic equivalent task hours per day, BMI – body mass index

*The regions mainly consuming sour pickled vegetables included Sichuan and Gansu. For continuous variables, BMI and waist circumference were described as mean (standard deviation), and others were described as median (interquartile range). Categorical variables were described as percentages.

During a mean follow-up of 11.7 (SD = 2.3) years and approximately 3.6 million person-years, 6348 GI cancer cases were documented, including 1166 oesophageal cancers, 2934 stomach cancers, and 2534 colorectal cancers, with corresponding incidence rates of 32.1, 80.8 and 69.8 per 100 000 person-years.

In the regions mainly consuming salted vegetables, preserved vegetable consumption was positively associated with stomach cancer in a dose-response manner (*P* for trend = 0.039), with multivariable-adjusted HR of 1.17 (95% CI = 1.00–1.37) for daily consumers compared with rare consumers. After correction for regression dilution bias, for each 50 g/d higher preserved vegetable consumption, the corresponding HR was 1.32 (95% CI = 1.05–1.67) for stomach cancer in the regions mainly consuming salted vegetables. No statistically significant associations between preserved vegetable consumption and other site-specific GI cancers were found in these regions (**Table 3**, Table S3 in the **Online Supplementary Document**).

**Table 3:** Adjusted hazard ratios for gastrointestinal tract cancers associated with preserved vegetable consumption in the regions mainly consuming salted vegetables (n = 202 927)*

Types of cancer	Frequency of preserved vegetable consumption					*P* for trend
	**Never**	**Monthly**	**1–3 day/week**	**4–6 day/week**	**Daily**	
**Oesophageal cancer**						
Case (incidence rate)	59 (19.8)	109 (19.4)	133 (20.6)	43 (22.7)	219 (30.9)	
*Model 1*	1.00	1.05 (0.75–1.47)	1.03 (0.75–1.42)	1.00 (0.67–1.49)	1.06 (0.79–1.42)	0.792
*Model 2*	1.00	1.07 (0.76–1.51)	1.08 (0.78–1.49)	1.05 (0.70–1.57)	1.05 (0.78–1.41)	0.930
**Stomach cancer**						
Case (incidence rate)	220 (74.0)	337 (60.1)	466 (72.1)	148 (78.3)	810 (114.5)	
*Model 1*	1.00	1.06 (0.89–1.27)	1.13 (0.96–1.33)	1.06 (0.85–1.31)	1.19 (1.02–1.39)	0.019
*Model 2*	1.00	1.05 (0.88–1.26)	1.14 (0.96–1.34)	1.06 (0.86–1.32)	1.17 (1.00–1.37)	0.039
**Colorectal cancer**						
Case (incidence rate)	287 (96.6)	421 (75.2)	467 (72.3)	154 (81.5)	659 (93.2)	
*Model 1*	1.00	0.97 (0.83–1.14)	0.92 (0.79–1.07)	1.00 (0.82–1.23)	0.99 (0.86–1.15)	0.877
*Model 2*	1.00	0.98 (0.84–1.16)	0.93 (0.80–1.09)	1.02 (0.83–1.25)	0.99 (0.85–1.14)	0.970
**Gastrointestinal tract cancers†**						
Case (incidence rate)	541 (182.6)	839 (150.0)	1023 (158.8)	322 (170.9)	1604 (227.6)	
*Model 1*	1.00	1.02 (0.91–1.15)	1.02 (0.91–1.13)	1.00 (0.87–1.15)	1.09 (0.98–1.20)	0.073
*Model 2*	1.00	1.03 (0.91–1.15)	1.03 (0.93–1.15)	1.02 (0.88–1.17)	1.08 (0.97–1.19)	0.145

*The regions mainly consuming salted vegetables included Qingdao, Harbin, Suzhou, and Zhejiang. Incidence rate was expressed as No./100 000 person-years. Model one stratified by age-at-risk (five-year bands), sex, and survey areas, and adjusted for education level, annual household income, and family history of cancers. Model two further adjusted for tobacco smoking, alcohol drinking, physical activity, BMI, waist circumference, and dietary factors (consumption of fresh vegetables, fresh fruits, meat, and spicy food).

†This endpoint is the first incident gastrointestinal tract cancer (which could be either oesophageal, stomach, or colorectal cancer).

In the regions mainly consuming sour pickled vegetables, after adjustment for sociodemographic factors, preserved vegetable consumption was significantly associated with an 18% (HR = 1.18; 95% CI = 1.01–1.39) higher risk of GI cancers when comparing daily consumers with rare consumers, with a significant linear trend (*P* for trend = 0.019). After further adjustment for lifestyle factors, the association was not significant (HR = 1.16; 95% CI = 0.98–1.36; *P* for trend = 0.072). Besides, a pronounced positive association was found for oesophageal cancer (*P* for trend = 0.013), with an adjusted HR of 1.35 (95% CI = 1.02–1.80) for daily consumers compared with rare consumers. After correction for regression dilution bias, for each 50 g/d higher preserved vegetable consumption, the corresponding HR was 1.73 (95% CI = 1.16–2.60) for oesophageal cancer in the regions mainly consuming sour pickled vegetables. For other site-specific GI cancers, there were no clear associations with preserved vegetable consumption in the regions mainly consuming sour pickled vegetables (**Table 4**, Table S3 in the **Online Supplementary Document**).

**Table 4:** Adjusted hazard ratios for gastrointestinal tract cancers associated with preserved vegetable consumption in the regions mainly consuming sour pickled vegetables (n = 105 372)*

Types of cancer	Frequency of preserved vegetable consumption					*P* for trend
	**Never**	**Monthly**	**1–3 day/week**	**4–6 day/week**	**Daily**	
**Oesophageal cancer**						
Case (incidence rate)	71 (58.5)	75 (38.4)	196 (37.8)	83 (44.5)	178 (85.6)	
*Model 1*	1.00	0.99 (0.71–1.38)	1.04 (0.78–1.38)	1.17 (0.84–1.62)	1.58 (1.20–2.09)	<0.001
*Model 2*	1.00	1.02 (0.73–1.42)	1.07 (0.80–1.42)	1.14 (0.82–1.58)	1.35 (1.02–1.80)	0.013
**Stomach cancer**						
Case (incidence rate)	121 (99.8)	136 (69.6)	391 (75.5)	155 (83.0)	150 (72.2)	
*Model 1*	1.00	0.79 (0.62–1.02)	0.84 (0.67–1.04)	0.84 (0.66–1.08)	0.87 (0.68–1.11)	0.561
*Model 2*	1.00	0.80 (0.62–1.03)	0.86 (0.69–1.07)	0.86 (0.66–1.10)	0.91 (0.71–1.16)	0.771
**Colorectal cancer**						
Case (incidence rate)	58 (47.8)	92 (47.1)	207 (40.0)	74 (39.6)	115 (55.3)	
*Model 1*	1.00	1.47 (1.05–2.05)	1.39 (1.03–1.88)	1.33 (0.93–1.89)	1.34 (0.97–1.84)	0.311
*Model 2*	1.00	1.53 (1.09–2.14)	1.45 (1.07–1.97)	1.35 (0.95–1.94)	1.37 (0.99–1.89)	0.292
**Gastrointestinal tract cancers†**						
Case (incidence rate*)*	238 (196.5)	291 (149.1)	767 (148.3)	304 (163.1)	419 (202.1)	
*Model 1*	1.00	1.01 (0.85–1.21)	1.04 (0.89–1.21)	1.07 (0.90–1.28)	1.18 (1.01–1.39)	0.019
*Model 2*	1.00	1.04 (0.87–1.24)	1.07 (0.92–1.25)	1.07 (0.90–1.28)	1.16 (0.98–1.36)	0.072

*The regions mainly consuming sour pickled vegetables included Sichuan and Gansu. Incidence rate was expressed as No./100 000 person-years. Model one stratified by age-at-risk (five-year bands), sex, and survey areas, and adjusted for education level, annual household income, and family history of cancers. Model two further adjusted for tobacco smoking, alcohol drinking, physical activity, BMI, waist circumference, and dietary factors (consumption of fresh vegetables, fresh fruits, meat, and spicy food).

†This endpoint is the first incident gastrointestinal tract cancer (which could be either oesophageal, stomach, or colorectal cancer).

Subgroup analyses were conducted for oesophageal and stomach cancer (Tables S4–S7 in the **Online Supplementary Document**). All subgroups yielded insignificant multiplicative interactions by the likelihood ratio tests (*P* for interaction > 0.05).

The associations did not substantially change in the various sensitivity analyses with further exclusions and adjustments (Table S8, Table S9 in the **Online Supplementary Document**).

## DISCUSSION

Based on the largest Chinese prospective cohort study, we collected preserved vegetable consumption information across geographically diverse regions. We found that daily consumption of salted vegetables was associated with 17% higher stomach cancer morbidity, and daily consumption of sour pickled vegetables was associated with a 35% increased risk of oesophageal cancer. Moreover, the above associations were independent of most known risk factors, such as tobacco smoking and alcohol drinking.

The present study revealed a significant positive association between salted vegetable consumption and stomach cancer, but not with sour pickled vegetable consumption. A hospital-based case-control study with a small sample in Yanting County, an area with a high incidence of stomach cancer, demonstrated that participants who consumed >227 g/week of salted vegetables had a 2.09 times higher risk of stomach cancer than never consumers, which was similar to our results. Nevertheless, the study also observed the positive association of weekly consuming sour pickled vegetables [15]. The Linxian general population trial cohort [9], by far the only cohort study conducted in an area traditionally consuming sour pickled vegetables [16], examined the association between preserved vegetables and stomach cancer risk in the general population of China. The study followed up adults aged 40–69 from 1984 to 2001 and found no statistically significant association between sour pickled vegetable consumption and stomach cancer, in line with our results [9]. Further validation of our study findings is warranted.

Meanwhile, our study revealed that sour pickled vegetable consumption was positively associated with oesophageal cancer, but not in the regions mainly consuming salted vegetables. A meta-analysis of case-control studies conducted in China found that both sour pickled vegetables (three studies) and salted vegetables (eight studies) were associated with an increased risk of oesophageal cancer [17]. Nevertheless, it is noteworthy that the heterogeneity among the eight studies of salted vegetables was significant. The Linxian cohort studies also researched the association between sour pickled vegetables and oesophageal cancer, and reported insignificant association after the adjustment for age and sex [9,10].

In the present study, we did not observe the associations of preserved vegetables with colorectal cancer. Previous studies reported a positive association of red meat and colorectal cancer, while inverse associations were observed between foods such as whole grains and colorectal cancer [18]. But limited evidence was established about the association between preserved vegetables and colorectal cancer. Two prospective studies among Japanese on colorectal cancer reported inconsistent results [19,20]. A cross-sectional study among Chinese reported a positive association between preserved vegetable consumption and colorectal polyp, a major risk factor for colorectal cancer [21]. However, our results did not support the association between preserved vegetables and colorectal cancer. The relatively less cases might partially explain the insignificant associations.

In summary, we observed a stronger association of sour pickled vegetable consumption with total GI cancers than salted vegetable consumption, and different types of preserved vegetable consumption were associated with different sites of GI cancers. Our findings suggested that different types of preserved vegetables influence the risks of total GI cancers and each site-specific GI cancer. Therefore, future studies on preserved vegetable consumption should be recommended to use more precise dietary measurements and collect specific cancer types.

Several mechanisms could link preserved vegetables and GI cancers. First, N-nitroso compounds, a well-documented carcinogen, were generated from the dietary nitrates and nitrites in green leafy and root vegetables by reacting with amines, amides, and other nitrosation precursors in the gastrointestinal tract [22]. N-nitroso compounds can damage the gastrointestinal barrier, promote cell proliferation, and increase DNA replication errors. Except for N-nitroso compounds, microorganisms in sour pickled vegetables could also produce potential carcinogens, such as Roussin red methyl ester and mycotoxins [23]. Second, the high salt concentration in salted vegetables may also play an essential role in developing stomach cancer [24,25]. Salt may directly cause inflammation to the gastric mucosa [26] and reduce gastric acid secretion, which could inhibit the synthesis of prostaglandin E, a protective substance in the gastric mucosa [27]. Additionally, salt may up-regulate the expression of the cytotoxin associated gene A (cagA) of *Helicobacter pylori*, a carcinogenic protein associated with gastric cancer [28].

In this large prospective study with more than 10-year follow-up, we examined the association between different types of preserved vegetable consumption and the risk of GI cancers. In addition, we utilised the repeat measurement of preserved vegetable consumption to correct the regression dilution bias. However, several limitations exist. First, we estimated the type of preserved vegetables based on the second resurvey, which may not accurately describe the intake for each participant, especially for those who consumed both types. However, few participants frequently consumed salted and sour pickled vegetables (4.1% for the regions mainly consuming salted vegetables, and 2.0% for the regions mainly consuming sour pickled vegetables). Second, we did not collect detailed information on the preservation process, such as ingredients and duration of preservation. Third, we did not adjust for total energy intake in the multivariate model due to the lack of information on the amount of consumption at baseline. However, BMI and waist circumference in the model can partially represent the level of energy intake. We also estimated daily energy intake using data from the resurvey and made additional adjustments in sensitivity analyses, which did not substantially change the results. Fourthly, although we adjusted for multiple potential confounding factors in the main and sensitivity analyses, residual confounding, such as the intake of other preserved foods, cannot be ruled out completely.

## CONCLUSIONS

Among Chinese adults, salted vegetable consumption was associated with a higher risk of stomach cancer, and sour pickled vegetable consumption was associated with a higher risk of oesophageal cancer. Our findings suggested that different types of preserved vegetables might have different effects on site-specific GI cancers, and controlling the intake of preserved vegetables might be beneficial for reducing the risk of GI cancers. Further research is needed to confirm the associations and potential mechanisms between different types of preserved vegetables and GI cancers.

## Additional material


Online Supplementary Document
