# Supplementary material for: Preserved vegetable consumption and gastrointestinal tract cancers: A prospective study
Source: J Glob Health. 2024 Nov 8;14:04191. doi: 10.7189/jogh.14.04191 (PMC11544521; doi:10.7189/jogh.14.04191)
Supplement: Online Supplementary Document [file jogh-14-04191-s001.pdf]

Supplementary material-Preserved vegetable consumption and gastrointestinal tract cancers: A prospective study

Contents

Members of the China Kadoorie Biobank collaborative group 1

Supplementary methods 3

Classification of 10 survey areas based on the type of preserved vegetables mainly consumed 3

Calculation of usual amount of preserved vegetable consumption 3

Table S1. Distribution of baseline multiclass characteristics in the regions mainly consuming salted vegetables (%) 6

Table S2. Distribution of baseline multiclass characteristics in the regions mainly consuming sour pickled vegetables (%) 9

Table S3. Correction for regression dilution bias (per 50 g/day) 12

Table S4. Subgroup analyses for the association between preserved vegetable consumption and oesophageal cancer in the regions mainly consuming salted vegetables (n=202,927) 13

Table S5. Subgroup analyses for the association between preserved vegetable consumption and stomach cancer in the regions mainly consuming salted vegetables (n=202,927) 15

Table S6. Subgroup analyses for the association between preserved vegetable consumption and oesophageal cancer in the regions mainly consuming sour pickled vegetables (n=105,372) 17

Table S7. Subgroup analyses for the association between preserved vegetable consumption and stomach cancer in the regions mainly consuming sour pickled vegetables (n=105,372) 19

Table S8. Sensitivity analyses for the association between preserved vegetable consumption and gastrointestinal tract cancers in the regions mainly consuming salted vegetables (n=202,927) 21

Table S9. Sensitivity analyses for the association between preserved vegetable consumption and gastrointestinal tract cancers in the regions mainly consuming sour pickled vegetables (n=105,372) 23

Figure S1. Participant flow chart **25**

Figure S2. The average intake level of salted and sour picked vegetables in the second resurvey (days/week) **26**

Figure S3. Frequency of preserved vegetable consumption in CKB study areas **27**

Reference **28**

Members of the China Kadoorie Biobank collaborative group

Members of the China Kadoorie Biobank collaborative group:

International Steering Committee: Junshi Chen, Zhengming Chen (PI), Robert Clarke, Rory Collins, Liming Li (PI), Jun Lv, Richard Peto, Robin Walters.

International Co-ordinating Centre, Oxford: Daniel Avery, Maxim Barnard, Derrick Bennett, Lazaros Belbasis, Ruth Boxall, Ka Hung Chan, Yiping Chen, Zhengming Chen, Charlotte Clarke, Johnathan Clarke; Robert Clarke, Huaidong Du, Ahmed Edris Mohamed, Hannah Fry, Simon Gilbert, Pek Kei Im, Andri Iona, Maria Kakkoura, Christiana Kartsonaki, Hubert Lam, Kuang Lin, James Liu, Mohsen Mazidi, Iona Millwood, Sam Morris, Qunhua Nie, Alfred Pozarickij, Maryanm Rahmati, Paul Ryder, Saredo Said, Dan Schmidt, Becky Stevens, Iain Turnbull, Robin Walters, Baihan Wang, Lin Wang, Neil Wright, Ling Yang, Xiaoming Yang, Pang Yao.

National Co-ordinating Centre, Beijing: Xiao Han, Can Hou, Qingmei Xia, Chao Liu, Jun Lv, Pei Pei, Dianjianyi Sun, Canqing Yu, Lang Pan.

10 Regional Co-ordinating Centres:

Qingdao CDC: Zengchang Pang, Ruqin Gao, Shanpeng Li, Haiping Duan, Shaojie Wang, Yongmei Liu, Ranran Du, Yajing Zang, Liang Cheng, Xiaocao Tian, Hua Zhang, Yaoming Zhai, Feng Ning, Xiaohui Sun, Feifei Li. **Licang CDC:** Silu Lv, Junzheng Wang, Wei Hou. **Heilongjiang Provincial CDC:** Wei Sun, Shichun Yan, Xiaoming Cui. **Nangang CDC:** Chi Wang, Zhenyuan Wu,Yanjie Li, Quan Kang. **Hainan Provincial CDC:** Huiming Luo, Tingting Ou. **Meilan CDC:** Xiangyang Zheng, Zhendong Guo, Shukuan Wu, Yilei Li, Huimei Li. **Jiangsu Provincial CDC:** Ming Wu, Yonglin Zhou, Jinyi Zhou, Ran Tao, Jie Yang, Jian Su. **Suzhou CDC:** Fang Liu, Jun Zhang, Yihe Hu, Yan Lu, Liangcai Ma, Aiyu Tang, Shuo Zhang, Jianrong Jin, Jingchao Liu. **Guangxi Provincial CDC:** Mei Lin, Zhenzhen Lu.

Liuzhou CDC: Lifang Zhou, Changping Xie, Jian Lan,Tingping Zhu,Yun Liu, Liuping Wei, Liyuan Zhou, Ningyu Chen, Yulu Qin, Sisi Wang. **Sichuan Provincial CDC:** Xianping Wu, Ningmei Zhang, Xiaofang Chen, Xiaoyu Chang. **Pengzhou CDC:** Mingqiang Yuan, Xia Wu, Xiaofang Chen, Wei Jiang, Jiaqiu Liu, Qiang Sun. **Gansu Provincial CDC:** Faqing Chen, Xiaolan Ren, Caixia Dong. **Maiji CDC:** Hui Zhang, Enke Mao, Xiaoping Wang, Tao Wang, Xi zhang. **Henan Provincial CDC:** Kai Kang, Shixian Feng, Huizi Tian, Lei Fan. **Huixian CDC:** XiaoLin Li, Huarong Sun, Pan He, Xukui Zhang. **Zhejiang Provincial CDC:** Min Yu, Ruying Hu, Hao Wang. **Tongxiang CDC**: Xiaoyi Zhang, Yuan Cao, Kaixu Xie, Lingli Chen, Dun Shen. **Hunan Provincial CDC:** Xiaojun Li, Donghui Jin, Li Yin, Huilin Liu, Zhongxi Fu. **Liuyang CDC:** Xin Xu, Hao Zhang, Jianwei Chen,Yuan Peng, Libo Zhang, Chan Qu.

Supplementary methods

Classification of 10 survey areas based on the type of preserved vegetables mainly consumed

The second resurvey of CKB study collected information on the intake frequency of two primary preserved vegetables in China, i.e., salted vegetables and sour pickled vegetables (n=25,041). The frequency levels included never/rarely, monthly, 1-3 days/week, 4-6 days/week, and daily, and we respectively assigned 0, 0.5, 2, 5, and 7 days/week to each frequency level for better quantification. Then, we estimated the average intake level (days/week) of each preserved vegetables by calculating the weighted sum of each frequency group, based on their respective percentages (Figure S1). Using 1 day/week as the demarcation criterion, we considered an average intake level of >1 day/week as habitual intake, and conversely for <1 day/week. As shown in Figure S1, the 10 survey areas were distinctly categorized into 3 groups: (1) Participants in 4 areas (Haikou, Liuzhou, Hunan, and Henan) consumed both major types of preserved vegetables <1 day/week, so we categorized them as the regions where people 'never/rarely consumed preserved vegetables'. (2) 4 other areas (Qingdao, Harbin, Suzhou, and Zhejiang) were categorized as the regions where people 'mainly consumed salted vegetables', as participants there consumed salted vegetables >1 day/week but sour pickled vegetables <1 day/week. (3) In contrast, participants in Sichuan and Gansu habitually consumed sour pickled vegetables but rarely consumed salted vegetables. Therefore, we classified the 2 areas as the regions where people 'mainly consumed sour pickled vegetables'.

Calculation of usual amount of preserved vegetable consumption

To correct regression dilution bias, we estimated usual amount of preserved vegetable intake (g/day) based on the frequency of preserved vegetable consumption in the baseline survey and the first resurvey, and the daily amount of salted and sour pickled vegetable

consumption in the second resurvey [1,2]. The steps of calculation were as follows.

1. Mean number of days per month of consumption (days/month) (variable D_n): The mean number of days per month assigned to each group was 0 for the 'never/rarely' group, 2.5 days for the 'monthly' group, 8.6 days for the '1-3 days/week' group, 21.5 days for the '4-6 days/week' group, and 30 days for the 'daily' group.

2. Daily amount of preserved vegetable consumption in the second resurvey (g/day): Firstly, at the second resurvey, monthly amount of total preserved vegetable consumption was approximately equal to the sum of monthly amount of salted vegetable and sour pickled vegetable consumption (g/month), which were calculated by multiplying daily amount and mean number of days per month consuming each preserved vegetables. Secondly, we estimated the total number of days per month of preserved vegetable consumption as the larger number of days per month of salted and sour pickled vegetable consumption. Thirdly, daily amount of total preserved vegetable consumption was equal to the quotient of monthly amount divided by total number of days per month of total preserved vegetable consumption.

3. Daily amount of preserved vegetable consumption at baseline (g/day) (variable B): Firstly, we stratified participants by age at baseline (<40, 40-59, or ≥60 years), 10 survey areas, sex, and baseline preserved vegetable consumption groups (i.e., 'never/rarely', 'monthly', '1-3 days/week', '4-6 days/week', or 'daily'). Secondly, the mean value of ***'daily amount of preserved vegetable consumption'*** for participants at the second resurvey in each stratum was used as a proxy of ***'baseline daily amount of preserved vegetable consumption'*** for baseline participants in the corresponding stratum**.** This process was based on the assumption that the daily amount of preserved vegetable consumption did not vary much from the baseline to the second resurvey (i.e., people may change their frequency of preserved vegetable consumption but the daily amount remains roughly the same).

4. Usual amount of preserved vegetable consumption per day (g/day) (Variable U_n):

The '***usual amount of preserved vegetable consumption per day***' for participants at n^{th} baseline group was estimated using the following formula:

- $$\mathbf{U_n = B \times \sum_{i=1}^{5}(D_n \times F_{ni}) / 30}$$

a) F_{ni} is the percentage of participants at the nth baseline frequency group switching to the ith frequency group at the first resurvey (see the table below). For example, for participants at the 'never/rarely' group in baseline survey, 48.6% remained at the 'never/rarely' group in the first resurvey, 28.1% switched to the 'monthly' group, 16.2% switched to the '1-3 days/week' group, 2.7% switched to the '4-6 days/week' group, and 4.3% switched to the 'daily' group.

Baseline frequency groups		First resurvey frequency groups n (F_{ni} / %)[a]				
		**Never/rarely 1**	**Monthly 2**	**1-3 days/week 3**	**4-6 days/week 4**	**Daily 5**
**1**	**Never/rarely**	1,708 (48.6)	989 (28.1)	571 (16.2)	96 (2.7)	151 (4.3)
**2**	**Monthly**	1,641 (26.1)	2,113 (33.6)	2,036 (32.4)	279 (4.4)	221 (3.5)
**3**	**1-3 days/week**	680 (12.9)	1,236 (23.5)	2,382 (45.2)	491 (9.3)	478 (9.1)
**4**	**4-6 days/week**	88 (7.5)	148 (12.5)	493 (41.7)	143 (12.1)	309 (26.2)
**5**	**Daily**	276 (8.1)	336 (9.8)	741 (21.7)	355 (10.4)	1,715 (50.1)

F_{ni} is the percentage of participants at the nth baseline frequency group switching to the ith frequency group in the first resurvey.

b) Therefore, the '***usual amount of preserved vegetable consumption per day***' for participants at 'never/rarely' group in baseline survey equal to **B** × (0 × 48.6% + 2.5 × 28.1% + 8.6 × 16.2% + 21.5 × 2.7% + 30 × 4.3%). The '***usual amount of preserved vegetable consumption per day***' for participants at other baseline group (i.e., 'monthly', '1-3 days/week', '4-6 days/week', or 'daily') were calculated using the same approach.

Table S1. Distribution of baseline multiclass characteristics in the regions mainly consuming salted vegetables (%)*

Characteristics	Frequency of preserved vegetable consumption					Overall
	Never	Monthly	1-3 days/week	4-6 days/week	Daily	
Number of participants	25,416	47,169	54,489	15,926	59,927	202,927
Education level						
No formal school	9.2	28.6	23.6	23.8	21.8	22.5
Primary school	16.3	28.2	25.5	25.9	24.0	24.5
Middle school	32.0	23.5	26.7	27.8	29.9	27.7
High school	26.3	12.9	16.1	15.3	16.9	16.8
College and above	16.2	6.8	8.1	7.3	7.4	8.5
Household income (yuan/year)						
<2,500	0.7	0.9	0.9	0.9	1.2	1.0
2,500-4,999	1.8	1.6	1.8	2.2	3.4	2.3
5,000-9,999	8.1	5.2	5.5	6.9	8.7	6.8
10,000-19,999	28.7	18.8	20.7	22.9	25.4	22.8
20,000-34,999	34.3	40.5	37.1	37.1	33.0	36.3
≥35,000	26.4	33.0	34.1	30.0	28.2	30.8
Smoking status in males						
Never/occasional smokers	30.5	22.1	24.4	23.5	19.8	23.2
Former smokers	10.0	9.3	8.2	6.8	8.4	8.6
Current and 1-9 cigarettes/day	10.7	8.9	9.1	9.6	11.4	10.0
Current and 10-19 cigarettes/day	18.9	18.7	19.7	21.1	21.6	20.0
Current and 20-29 cigarettes/day	22.0	31.0	29.1	30.3	29.2	28.8
Current and ≥30 cigarettes/day	7.9	10.0	9.5	8.7	9.6	9.4
Smoking status in females						
Never/occasional smokers	96.3	97.5	97.4	96.4	96.9	96.8

Characteristics	Frequency of preserved vegetable consumption					Overall
	Never	Monthly	1-3 days/week	4-6 days/week	Daily	
Former smokers	0.7	0.4	0.4	0.5	0.4	0.5
Current and 1-9 cigarettes/day	1.7	1.4	1.2	1.6	1.4	1.6
Current and 10-19 cigarettes/day	0.9	0.5	0.7	1.0	0.9	0.8
Current and 20-29 cigarettes/day	0.3	0.2	0.3	0.4	0.4	0.3
Current and ≥30 cigarettes/day	0.1	0.0	0.0	0.1	0.0	0.0
Alcohol consumption in males						
Not weekly drinking	50.6	53.3	50.2	47.8	40.6	48.0
Ex-regular drinkers	9.5	7.6	7.3	7.3	9.1	8.2
Weekly drinkers	16.7	12.7	15.1	16.2	17.1	15.4
Daily and <15 g/day	1.1	0.9	1.0	0.8	0.9	1.0
Daily and 15-29 g/day	5.7	4.8	5.8	6.5	6.7	5.9
Daily and 30-59 g/day	8.6	9.2	10.1	10.7	13.3	10.6
Daily and ≥60 g/day	7.8	11.5	10.5	10.7	12.3	10.9
Alcohol consumption in females						
Not weekly drinking	96.7	97.7	97.7	97.4	97.3	97.4
Ex-regular drinkers	0.6	0.5	0.4	0.4	0.4	0.5
Weekly drinkers	1.9	1.2	1.3	1.5	1.6	1.5
Daily and <15 g/day	0.5	0.3	0.2	0.3	0.3	0.3
Daily and 15-29 g/day	0.2	0.2	0.2	0.3	0.3	0.2
Daily and 30-59 g/day	0.1	0.1	0.2	0.1	0.1	0.1
Daily and ≥60 g/day	0.0	0.0	0.0	0.0	0.0	0.0
Fresh fruit consumption						
Never/rarely	6.2	2.2	3.1	3.4	5.4	4.0
Monthly	13.0	35.9	23.3	22.8	22.5	24.7

Characteristics	Frequency of preserved vegetable consumption					Overall
	Never	Monthly	1-3 days/week	4-6 days/week	Daily	
1-3 days/week	22.9	31.2	35.8	32.0	30.3	31.2
4-6 days/week	8.2	9.2	10.5	18.9	7.1	9.5
Daily	49.7	21.5	27.3	22.9	34.7	30.6
Meat consumption						
Never/rarely	3.4	1.0	1.4	1.7	2.5	1.9
Monthly	6.8	6.3	5.4	5.5	6.5	6.1
1-3 days/week	31.0	39.6	37.8	37.0	37.1	37.1
4-6 days/week	12.0	25.8	21.6	29.2	12.0	19.1
Daily	46.8	27.3	33.8	26.6	41.9	35.8
Spicy food consumption						
Never/rarely	46.2	54.4	45.8	42.8	40.7	46.1
Monthly	34.6	30.8	33.1	31.6	30.6	31.9
1-2 days/week	8.0	6.2	9.3	11.3	10.2	8.8
3-5 days/week	6.4	4.9	7.4	9.3	9.3	7.4
Daily/almost every day	4.8	3.7	4.4	5.0	9.2	5.8

*The regions mainly consuming salted vegetables included Qingdao, Harbin, Suzhou, and Zhejiang.

Table S2. Distribution of baseline multiclass characteristics in the regions mainly consuming sour pickled vegetables (%)*

Characteristics	Frequency of preserved vegetable consumption					Overall
	Never	Monthly	1-3 days/week	4-6 days/week	Daily	
Number of participants	10,621	16,575	44,886	16,134	17,156	105,372
Education level						
No formal school	23.8	29.4	32.4	38.6	19.0	29.8
Primary school	46.2	37.3	37.0	35.7	45.3	39.1
Middle school	21.8	23.3	21.8	19.2	27.5	22.6
High school	6.0	8.1	7.4	5.5	6.3	6.9
College and above	2.2	1.9	1.4	1.0	1.9	1.6
Household income (yuan/year)						
<2,500	14.0	5.9	5.5	5.4	9.3	7.0
2,500-4,999	20.0	21.3	18.9	23.2	17.8	19.9
5,000-9,999	34.3	44.1	45.9	46.9	36.5	43.1
10,000-19,999	24.2	22.5	25.2	20.3	27.3	24.2
20,000-34,999	4.6	4.0	3.2	3.0	6.1	3.9
≥35,000	2.9	2.2	1.3	1.2	3.0	1.9
Smoking status in males						
Never/occasional smokers	21.9	22.7	22.7	20.9	17.9	21.5
Former smokers	4.0	3.4	3.5	3.4	4.3	3.7
Current and 1-9 cigarettes/day	12.6	16.5	17.8	20.7	12.2	16.5
Current and 10-19 cigarettes/day	21.4	23.1	23.0	25.6	20.5	22.8
Current and 20-29 cigarettes/day	22.6	23.0	22.5	19.3	26.5	22.8
Current and ≥30 cigarettes/day	17.5	11.3	10.5	10.1	18.6	12.7
Smoking status in females						
Never/occasional smokers	85.2	92.9	95.1	94.7	87.8	92.5

Characteristics	Frequency of preserved vegetable consumption					Overall
	Never	Monthly	1-3 days/week	4-6 days/week	Daily	
Former smokers	1.5	0.6	0.4	0.5	1.2	0.7
Current and 1-9 cigarettes/day	7.4	3.8	2.5	2.6	5.8	3.7
Current and 10-19 cigarettes/day	3.8	1.8	1.3	1.4	3.3	2.0
Current and 20-29 cigarettes/day	1.6	0.8	0.6	0.7	1.7	0.9
Current and ≥30 cigarettes/day	0.5	0.1	0.1	0.1	0.2	0.2
Alcohol consumption in males						
Not weekly drinking	49.5	65.3	70.1	70.4	32.8	60.9
Ex-regular drinkers	18.6	9.1	6.6	6.4	11.5	9.0
Weekly drinkers	8.5	8.8	7.9	6.6	10.2	8.3
Daily and <15 g/day	0.4	0.3	0.3	0.1	0.3	0.3
Daily and 15-29 g/day	1.7	1.6	1.5	1.3	2.8	1.7
Daily and 30-59 g/day	5.8	3.8	3.4	3.7	9.3	4.8
Daily and ≥60 g/day	15.5	11.1	10.2	11.5	33.1	15.0
Alcohol consumption in females						
Not weekly drinking	91.6	95.7	96.5	96.4	88.6	94.6
Ex-regular drinkers	4.1	1.8	1.3	1.3	3.7	2.0
Weekly drinkers	1.7	1.0	0.8	1.0	2.4	1.2
Daily and <15 g/day	0.3	0.2	0.1	0.1	0.5	0.2
Daily and 15-29 g/day	0.8	0.5	0.5	0.5	1.7	0.8
Daily and 30-59 g/day	0.9	0.5	0.5	0.4	2.0	0.8
Daily and ≥60 g/day	0.6	0.3	0.3	0.3	1.1	0.4
Fresh fruit consumption						
Never/rarely	14.3	5.2	4.7	8.7	8.7	7.0
Monthly	28.1	48.8	39.2	41.5	25.5	37.7

Characteristics	Frequency of preserved vegetable consumption					Overall
	Never	Monthly	1-3 days/week	4-6 days/week	Daily	
1-3 days/week	36.4	29.5	39.5	30.2	37.9	36.0
4-6 days/week	10.2	9.7	11.2	14.1	11.2	11.3
Daily	11.0	6.8	5.4	5.5	16.7	8.0
Meat consumption						
Never/rarely	4.7	7.4	8.3	19.7	4.0	8.8
Monthly	8.0	25.2	25.0	23.2	6.3	20.0
1-3 days/week	50.1	40.0	42.3	31.6	39.9	40.7
4-6 days/week	16.7	14.0	13.1	13.9	19.6	14.8
Daily	20.5	13.4	11.3	11.6	30.2	15.7
Spicy food consumption						
Never/rarely	17.2	9.8	8.7	10.2	7.5	9.8
Monthly	17.6	17.7	18.1	17.9	9.6	16.6
1-2 days/week	5.9	9.8	9.3	7.9	4.4	8.0
3-5 days/week	5.4	8.0	9.9	8.6	4.1	8.0
Daily/almost every day	53.9	54.7	54.0	55.4	74.4	57.6

*The regions mainly consuming sour pickled vegetables included Sichuan and Gansu.

Table S3. Correction for regression dilution bias (per 50 g/day)*

**Types of cancer**	**Salted vegetables (n=202,927)**	**Sour pickled vegetables (n=105,372)**
Oesophageal cancer	1.01 (0.65, 1.59)	1.73 (1.16, 2.60)
Stomach cancer	1.32 (1.05, 1.67)	1.03 (0.71, 1.51)
Colorectal cancer	1.02 (0.82, 1.28)	1.14 (0.72, 1.83)
Gastrointestinal tract cancers†	1.15 (0.98, 1.34)	1.27 (0.99, 1.62)

*The results were conducted based on model 2. The regions mainly consuming salted vegetables included Qingdao, Harbin, Suzhou, and Zhejiang. The regions mainly consuming sour pickled vegetables included Sichuan and Gansu.

†This endpoint is the first incident gastrointestinal tract cancer (which could be either oesophageal, stomach, or colorectal cancer).

Table S4. Subgroup analyses for the association between preserved vegetable consumption and oesophageal cancer in the regions mainly consuming salted vegetables (n=202,927)*

Subgroups	Frequency of preserved vegetable consumption					*P* for interaction
	Never	Monthly	1-3 days/week	4-6 days/week	Daily	
Age						0.924
<60 years	1.00	0.98 (0.59, 1.65)	0.97 (0.60, 1.58)	1.05 (0.59, 1.88)	1.03 (0.66, 1.61)	
≥60 years	1.00	1.15 (0.73, 1.83)	1.19 (0.77, 1.82)	1.02 (0.58, 1.81)	1.05 (0.70, 1.57)	
Sex						0.078
Males	1.00	1.23 (0.84, 1.80)	1.12 (0.78, 1.61)	0.95 (0.59, 1.54)	1.14 (0.82, 1.61)	
Females	1.00	0.57 (0.25, 1.28)	0.96 (0.49, 1.89)	1.24 (0.58, 2.67)	0.78 (0.42, 1.46)	
Healthy body shape†						0.791
No	1.00	1.08 (0.58, 2.02)	1.08 (0.61, 1.91)	1.25 (0.62, 2.54)	1.15 (0.69, 1.93)	
Yes	1.00	1.06 (0.70, 1.61)	1.08 (0.73, 1.60)	0.97 (0.59, 1.59)	1.01 (0.70, 1.46)	
Tobacco smoking in males‡						0.758
Never-regular smokers	1.00	1.63 (0.55, 4.86)	1.31 (0.45, 3.77)	1.01 (0.23, 4.42)	0.68 (0.22, 2.08)	
Ever-regular smokers	1.00	1.17 (0.78, 1.77)	1.10 (0.75, 1.63)	0.93 (0.56, 1.56)	1.18 (0.82, 1.68)	
Alcohol drinking in males‡						0.537
Never-regular drinkers	1.00	1.22 (0.69, 2.13)	1.10 (0.64, 1.88)	1.00 (0.50, 2.01)	0.90 (0.54, 1.50)	
Ever-regular drinkers	1.00	1.21 (0.71, 2.04)	1.11 (0.67, 1.82)	0.83 (0.42, 1.62)	1.25 (0.80, 1.97)	
Spicy food consumption						0.446
Not daily	1.00	1.13 (0.79, 1.61)	1.14 (0.82, 1.59)	1.06 (0.70, 1.61)	1.06 (0.78, 1.45)	
Daily	1.00	0.38 (0.08, 1.68)	0.23 (0.04, 1.20)	0.75 (0.15, 3.79)	0.74 (0.24, 2.24)	
Tea drinking						0.362
Less than daily/daily at warm tea	1.00	1.07 (0.70, 1.65)	1.01 (0.67, 1.51)	1.13 (0.70, 1.83)	0.93 (0.63, 1.35)	
Daily at hot/burning hot tea	1.00	1.05 (0.60, 1.86)	1.19 (0.71, 2.02)	0.83 (0.39, 1.75)	1.26 (0.77, 2.05)	
Fresh fruit consumption						0.845
<4 days/week	1.00	1.07 (0.72, 1.59)	1.04 (0.72, 1.52)	0.93 (0.57, 1.50)	0.96 (0.67, 1.36)	

Subgroups	Frequency of preserved vegetable consumption					*P* for interaction
	Never	Monthly	1-3 days/week	4-6 days/week	Daily	
≥4 days/week	1.00	0.99 (0.48, 2.03)	1.16 (0.62, 2.16)	1.34 (0.63, 2.86)	1.30 (0.74, 2.29)	
Meat consumption						0.096
<4 days/week	1.00	0.84 (0.51, 1.39)	1.10 (0.69, 1.73)	1.28 (0.74, 2.19)	0.95 (0.62, 1.46)	
≥4 days/week	1.00	1.37 (0.86, 2.18)	1.06 (0.68, 1.67)	0.79 (0.42, 1.50)	1.19 (0.79, 1.79)	

*The regions mainly consuming salted vegetables included Qingdao, Harbin, Suzhou, and Zhejiang.

†Healthy body shape was defined according to BMI and waist circumference. Participants having BMI <18.5 or ≥28.0 kg/m^2 or having waist circumference ≥90 cm (males)/85 cm (females) were considered as high risk, which emphasized avoidance of extremely high or low weight and abdominal obesity.

‡Subgroup analyses for tobacco smoking and alcohol drinking were conducted among males due to the small number of ever-regular smokers and ever-regular drinkers among females.

Table S5. Subgroup analyses for the association between preserved vegetable consumption and stomach cancer in the regions mainly consuming salted vegetables (n=202,927)*

Subgroups	Frequency of preserved vegetable consumption					*P* for interaction
	Never	Monthly	1-3 days/week	4-6 days/week	Daily	
Age						0.672
<60 years	1.00	0.96 (0.74, 1.24)	1.11 (0.88, 1.41)	0.96 (0.71, 1.30)	1.08 (0.86, 1.36)	
≥60 years	1.00	1.15 (0.90, 1.48)	1.15 (0.91, 1.46)	1.17 (0.87, 1.59)	1.25 (1.01, 1.55)	
Sex						0.295
Males	1.00	0.97 (0.78, 1.21)	1.08 (0.89, 1.32)	1.07 (0.82, 1.38)	1.07 (0.88, 1.28)	
Females	1.00	1.26 (0.92, 1.74)	1.26 (0.93, 1.70)	1.09 (0.74, 1.61)	1.45 (1.10, 1.92)	
Healthy body shape†						0.306
No	1.00	1.11 (0.83, 1.49)	0.95 (0.72, 1.26)	0.95 (0.65, 1.37)	1.15 (0.90, 1.48)	
Yes	1.00	1.05 (0.83, 1.32)	1.24 (1.01, 1.53)	1.13 (0.87, 1.47)	1.20 (0.98, 1.47)	
Tobacco smoking in males‡						0.905
Never-regular smokers	1.00	0.91 (0.51, 1.64)	1.27 (0.77, 2.10)	1.02 (0.49, 2.11)	1.03 (0.62, 1.70)	
Ever-regular smokers	1.00	0.98 (0.77, 1.24)	1.06 (0.85, 1.31)	1.07 (0.81, 1.40)	1.07 (0.87, 1.31)	
Alcohol drinking in males‡						0.378
Never-regular drinkers	1.00	0.99 (0.74, 1.31)	1.03 (0.79, 1.33)	1.16 (0.83, 1.61)	0.95 (0.74, 1.22)	
Ever-regular drinkers	1.00	0.98 (0.69, 1.38)	1.18 (0.86, 1.62)	0.96 (0.64, 1.45)	1.24 (0.93, 1.67)	
Spicy food consumption						0.503
Not daily	1.00	1.05 (0.87, 1.26)	1.11 (0.94, 1.32)	1.06 (0.85, 1.32)	1.16 (0.99, 1.36)	
Daily	1.00	1.32 (0.50, 3.47)	2.05 (0.87, 4.84)	1.26 (0.41, 3.89)	1.54 (0.69, 3.43)	
Tea drinking						0.122
Less than daily/daily at warm tea	1.00	1.14 (0.92, 1.42)	1.22 (1.00, 1.49)	1.21 (0.94, 1.56)	1.31 (1.08, 1.58)	
Daily at hot/burning hot tea	1.00	0.90 (0.65, 1.24)	0.99 (0.74, 1.33)	0.78 (0.52, 1.17)	0.94 (0.72, 1.24)	
Fresh fruit consumption						0.918
<4 days/week	1.00	1.14 (0.90, 1.44)	1.22 (0.98, 1.53)	1.12 (0.85, 1.48)	1.23 (0.99, 1.51)	

Subgroups	Frequency of preserved vegetable consumption					P for interaction
	Never	Monthly	1-3 days/week	4-6 days/week	Daily	
≥4 days/week	1.00	0.96 (0.72, 1.28)	1.03 (0.80, 1.34)	1.01 (0.72, 1.43)	1.11 (0.88, 1.40)	
Meat consumption						0.377
<4 days/week	1.00	0.92 (0.70, 1.22)	1.13 (0.88, 1.45)	1.11 (0.81, 1.51)	1.15 (0.91, 1.46)	
≥4 days/week	1.00	1.17 (0.93, 1.49)	1.15 (0.92, 1.44)	1.02 (0.76, 1.38)	1.20 (0.98, 1.48)	

*The regions mainly consuming salted vegetables included Qingdao, Harbin, Suzhou, and Zhejiang.

†Healthy body shape was defined according to BMI and waist circumference. Participants having BMI <18.5 or ≥28.0 kg/m^2 or having waist circumference ≥90 cm (males)/85 cm (females) were considered as high risk, which emphasized avoidance of extremely high or low weight and abdominal obesity.

‡Subgroup analyses for tobacco smoking and alcohol drinking were conducted among males due to the small number of ever-regular smokers and ever-regular drinkers among females.

Table S6. Subgroup analyses for the association between preserved vegetable consumption and oesophageal cancer in the regions mainly consuming sour pickled vegetables (n=105,372)*

Subgroups	Frequency of preserved vegetable consumption					*P* for interaction
	Never	Monthly	1-3 days/week	4-6 days/week	Daily	
Age						0.827
<60 years	1.00	0.92 (0.57, 1.50)	1.08 (0.72, 1.63)	1.02 (0.63, 1.65)	1.32 (0.88, 1.97)	
≥60 years	1.00	1.13 (0.71, 1.78)	1.05 (0.70, 1.57)	1.29 (0.82, 2.03)	1.37 (0.91, 2.05)	
Sex						0.246
Males	1.00	1.20 (0.83, 1.74)	1.15 (0.83, 1.59)	1.19 (0.81, 1.73)	1.46 (1.06, 2.01)	
Females	1.00	0.53 (0.24, 1.17)	0.87 (0.47, 1.61)	1.02 (0.51, 2.03)	1.06 (0.55, 2.04)	
Healthy body shape†						0.952
No	1.00	0.65 (0.30, 1.38)	0.79 (0.42, 1.48)	0.92 (0.45, 1.88)	1.22 (0.65, 2.29)	
Yes	1.00	1.12 (0.77, 1.62)	1.13 (0.82, 1.56)	1.18 (0.81, 1.71)	1.38 (1.01, 1.90)	
Tobacco smoking in males‡						0.966
Never-regular smokers	1.00	1.36 (0.27, 6.87)	1.58 (0.39, 6.36)	1.00 (0.17, 5.87)	1.51 (0.36, 6.28)	
Ever-regular smokers	1.00	1.20 (0.82, 1.76)	1.13 (0.81, 1.58)	1.19 (0.81, 1.76)	1.45 (1.04, 2.01)	
Alcohol drinking in males‡						0.665
Never-regular drinkers	1.00	1.11 (0.65, 1.91)	1.08 (0.67, 1.76)	0.98 (0.56, 1.73)	1.18 (0.69, 2.02)	
Ever-regular drinkers	1.00	1.26 (0.75, 2.11)	1.18 (0.75, 1.84)	1.36 (0.81, 2.29)	1.60 (1.05, 2.43)	
Spicy food consumption						0.495
Not daily	1.00	0.78 (0.48, 1.28)	0.99 (0.66, 1.48)	1.10 (0.68, 1.76)	1.18 (0.76, 1.85)	
Daily	1.00	1.33 (0.84, 2.13)	1.16 (0.77, 1.75)	1.22 (0.76, 1.96)	1.53 (1.03, 2.26)	
Tea drinking						0.276
Less than daily/daily at warm tea	1.00	0.86 (0.57, 1.30)	0.96 (0.68, 1.37)	0.95 (0.62, 1.44)	1.47 (1.04, 2.07)	
Daily at hot/burning hot tea	1.00	1.45 (0.81, 2.58)	1.27 (0.77, 2.11)	1.49 (0.85, 2.60)	1.14 (0.68, 1.91)	
Fresh fruit consumption						0.093
<4 days/week	1.00	1.20 (0.84, 1.71)	1.18 (0.86, 1.61)	1.24 (0.86, 1.78)	1.47 (1.07, 2.01)	

Subgroups	Frequency of preserved vegetable consumption					*P* for interaction
	Never	Monthly	1-3 days/week	4-6 days/week	Daily	
≥4 days/week	1.00	0.22 (0.06, 0.79)	0.53 (0.25, 1.13)	0.60 (0.25, 1.46)	0.72 (0.36, 1.45)	
Meat consumption						0.918
<4 days/week	1.00	1.04 (0.68, 1.59)	1.03 (0.71, 1.50)	1.10 (0.72, 1.69)	1.45 (0.98, 2.14)	
≥4 days/week	1.00	0.95 (0.54, 1.64)	1.16 (0.74, 1.81)	1.19 (0.70, 2.00)	1.28 (0.84, 1.95)	

*The regions mainly consuming sour pickled vegetables included Sichuan and Gansu.

†Healthy body shape was defined according to BMI and waist circumference. Participants having BMI <18.5 or ≥28.0 kg/m^2 or having waist circumference ≥90 cm (males)/85 cm (females) were considered as high risk, which emphasized avoidance of extremely high or low weight and abdominal obesity.

‡Subgroup analyses for tobacco smoking and alcohol drinking were conducted among males due to the small number of ever-regular smokers and ever-regular drinkers among females.

Table S7. Subgroup analyses for the association between preserved vegetable consumption and stomach cancer in the regions mainly consuming sour pickled vegetables (n=105,372)*

Subgroups	Frequency of preserved vegetable consumption					*P* for interaction
	Never	Monthly	1-3 days/week	4-6 days/week	Daily	
Age						0.449
<60 years	1.00	0.71 (0.49, 1.04)	0.75 (0.54, 1.04)	0.76 (0.52, 1.11)	0.92 (0.65, 1.31)	
≥60 years	1.00	0.89 (0.63, 1.26)	0.99 (0.73, 1.33)	0.96 (0.68, 1.35)	0.88 (0.62, 1.24)	
Sex						0.914
Males	1.00	0.85 (0.62, 1.16)	0.90 (0.69, 1.19)	0.92 (0.68, 1.26)	1.00 (0.74, 1.36)	
Females	1.00	0.70 (0.46, 1.09)	0.78 (0.53, 1.14)	0.74 (0.47, 1.14)	0.74 (0.48, 1.14)	
Healthy body shape†						0.778
No	1.00	0.85 (0.50, 1.43)	1.00 (0.64, 1.57)	1.00 (0.60, 1.66)	0.85 (0.51, 1.44)	
Yes	1.00	0.79 (0.59, 1.06)	0.82 (0.64, 1.06)	0.81 (0.60, 1.08)	0.92 (0.69, 1.21)	
Tobacco smoking in males‡						0.296
Never-regular smokers	1.00	1.22 (0.48, 3.11)	0.68 (0.28, 1.63)	1.09 (0.41, 2.90)	1.11 (0.42, 2.94)	
Ever-regular smokers	1.00	0.83 (0.60, 1.17)	0.94 (0.70, 1.25)	0.91 (0.65, 1.26)	1.02 (0.74, 1.39)	
Alcohol drinking in males‡						0.060
Never-regular drinkers	1.00	0.90 (0.63, 1.30)	0.91 (0.66, 1.25)	0.81 (0.56, 1.17)	0.86 (0.58, 1.27)	
Ever-regular drinkers	1.00	0.66 (0.33, 1.31)	0.90 (0.53, 1.52)	1.43 (0.80, 2.55)	1.17 (0.71, 1.94)	
Spicy food consumption						0.393
Not daily	1.00	1.00 (0.71, 1.41)	0.96 (0.70, 1.32)	0.89 (0.62, 1.27)	0.96 (0.65, 1.40)	
Daily	1.00	0.62 (0.42, 0.91)	0.77 (0.57, 1.06)	0.84 (0.59, 1.20)	0.83 (0.60, 1.16)	
Tea drinking						0.565
Less than daily/daily at warm tea	1.00	0.87 (0.65, 1.16)	0.86 (0.66, 1.11)	0.85 (0.63, 1.15)	0.88 (0.66, 1.17)	
Daily at hot/burning hot tea	1.00	0.63 (0.37, 1.08)	0.84 (0.54, 1.31)	0.85 (0.52, 1.39)	0.93 (0.57, 1.52)	
Fresh fruit consumption						0.263
<4 days/week	1.00	0.87 (0.66, 1.14)	0.89 (0.70, 1.14)	0.92 (0.70, 1.22)	0.94 (0.71, 1.23)	

Subgroups	Frequency of preserved vegetable consumption					*P* for interaction
	Never	Monthly	1-3 days/week	4-6 days/week	Daily	
≥4 days/week	1.00	0.46 (0.22, 0.96)	0.68 (0.39, 1.19)	0.56 (0.28, 1.09)	0.71 (0.40, 1.25)	
Meat consumption						0.706
<4 days/week	1.00	0.85 (0.63, 1.14)	0.87 (0.67, 1.13)	0.83 (0.62, 1.13)	0.98 (0.72, 1.33)	
≥4 days/week	1.00	0.66 (0.40, 1.10)	0.89 (0.59, 1.33)	0.96 (0.60, 1.55)	0.82 (0.54, 1.24)	

*The regions mainly consuming sour pickled vegetables included Sichuan and Gansu.

†Healthy body shape was defined according to BMI and waist circumference. Participants having BMI <18.5 or ≥28.0 kg/m^2 or having waist circumference ≥90 cm (males)/85 cm (females) were considered as high risk, which emphasized avoidance of extremely high or low weight and abdominal obesity.

‡Subgroup analyses for tobacco smoking and alcohol drinking were conducted among males due to the small number of ever-regular smokers and ever-regular drinkers among females.

Table S8. Sensitivity analyses for the association between preserved vegetable consumption and gastrointestinal tract cancers in the regions mainly consuming salted vegetables (n=202,927)*

Types of cancer	Frequency of preserved vegetable consumption					*P* for trend
	Never	Monthly	1-3 days/week	4-6 days/week	Daily	
**Oesophageal cancer**						
Model 2	1.00	1.07 (0.76, 1.51)	1.08 (0.78, 1.49)	1.05 (0.70, 1.57)	1.05 (0.78, 1.41)	0.930
Excluding cases in the first 2 years of follow-up	1.00	1.01 (0.70, 1.46)	0.99 (0.70, 1.40)	0.96 (0.62, 1.49)	1.00 (0.73, 1.37)	0.340
Additionally adjusted daily energy intake	1.00	1.07 (0.76, 1.51)	1.08 (0.78, 1.49)	1.05 (0.70, 1.57)	1.05 (0.78, 1.41)	0.907
Additionally adjusted intake of dietary supplements†	1.00	1.08 (0.76, 1.51)	1.08 (0.79, 1.49)	1.05 (0.70, 1.57)	1.05 (0.78, 1.41)	0.924
Additionally adjusted the years of owning a refrigerator	1.00	1.07 (0.76, 1.51)	1.08 (0.78, 1.49)	1.04 (0.70, 1.56)	1.04 (0.77, 1.40)	0.964
Additionally adjusted other food groups consumption‡	1.00	1.09 (0.77, 1.54)	1.08 (0.78, 1.49)	1.04 (0.69, 1.57)	1.05 (0.78, 1.41)	0.977
Additionally adjusted tea drinking§	1.00	1.08 (0.77, 1.52)	1.08 (0.79, 1.49)	1.05 (0.70, 1.58)	1.05 (0.78, 1.41)	0.943
**Stomach cancer**						
Model 2	1.00	1.05 (0.88, 1.26)	1.14 (0.96, 1.34)	1.06 (0.86, 1.32)	1.17 (1.00, 1.37)	0.039
Excluding cases in the first 2 years of follow-up	1.00	1.07 (0.88, 1.30)	1.17 (0.98, 1.40)	1.08 (0.85, 1.36)	1.23 (1.04, 1.45)	0.004
Additionally adjusted daily energy intake	1.00	1.06 (0.88, 1.27)	1.14 (0.96, 1.34)	1.06 (0.86, 1.32)	1.17 (1.00, 1.37)	0.045
Additionally adjusted intake of dietary supplements†	1.00	1.05 (0.88, 1.26)	1.14 (0.96, 1.34)	1.06 (0.86, 1.32)	1.17 (1.00, 1.37)	0.039
Additionally adjusted the years of owning a refrigerator	1.00	1.05 (0.88, 1.26)	1.14 (0.96, 1.34)	1.06 (0.86, 1.32)	1.17 (1.00, 1.37)	0.042
Additionally adjusted other food groups consumption‡	1.00	1.04 (0.86, 1.24)	1.12 (0.95, 1.33)	1.05 (0.85, 1.30)	1.18 (1.01, 1.38)	0.027
Additionally adjusted tea drinking§	1.00	1.05 (0.88, 1.26)	1.13 (0.96, 1.34)	1.06 (0.86, 1.31)	1.17 (1.00, 1.37)	0.038
**Colorectal cancer**						
Model 2	1.00	0.98 (0.84, 1.16)	0.93 (0.80, 1.09)	1.02 (0.83, 1.25)	0.99 (0.85, 1.14)	0.970
Excluding cases in the first 2 years of follow-up	1.00	1.00 (0.84, 1.19)	0.94 (0.80, 1.10)	1.02 (0.82, 1.26)	0.98 (0.84, 1.14)	0.999
Additionally adjusted daily energy intake	1.00	0.98 (0.84, 1.16)	0.93 (0.80, 1.09)	1.02 (0.83, 1.25)	0.99 (0.85, 1.14)	0.999
Additionally adjusted intake of dietary supplements†	1.00	0.99 (0.84, 1.16)	0.94 (0.80, 1.09)	1.02 (0.84, 1.25)	0.99 (0.86, 1.14)	0.996
Additionally adjusted the years of owning a refrigerator	1.00	0.98 (0.84, 1.16)	0.93 (0.80, 1.09)	1.02 (0.84, 1.25)	0.99 (0.86, 1.14)	0.982
Additionally adjusted other food groups consumption‡	1.00	0.96 (0.81, 1.13)	0.91 (0.78, 1.07)	1.00 (0.81, 1.22)	0.98 (0.85, 1.14)	0.946
Additionally adjusted tea drinking§	1.00	0.99 (0.84, 1.16)	0.93 (0.80, 1.09)	1.02 (0.83, 1.25)	0.99 (0.85, 1.14)	0.967

Types of cancer	Frequency of preserved vegetable consumption					*P* for trend
	Never	Monthly	1-3 days/week	4-6 days/week	Daily	
**Gastrointestinal tract cancers‖**						
Model 2	1.00	1.03 (0.91, 1.15)	1.03 (0.93, 1.15)	1.02 (0.88, 1.17)	1.08 (0.97, 1.19)	0.145
Excluding cases in the first 2 years of follow-up	1.00	1.04 (0.92, 1.18)	1.03 (0.92, 1.16)	1.02 (0.88, 1.19)	1.09 (0.98, 1.22)	0.112
Additionally adjusted daily energy intake	1.00	1.03 (0.92, 1.15)	1.03 (0.93, 1.15)	1.02 (0.88, 1.17)	1.08 (0.97, 1.19)	0.154
Additionally adjusted intake of dietary supplements†	1.00	1.03 (0.92, 1.16)	1.03 (0.93, 1.15)	1.02 (0.88, 1.17)	1.08 (0.97, 1.19)	0.139
Additionally adjusted the years of owning a refrigerator	1.00	1.03 (0.91, 1.15)	1.03 (0.93, 1.15)	1.02 (0.88, 1.17)	1.08 (0.97, 1.19)	0.142
Additionally adjusted other food groups consumption‡	1.00	1.01 (0.90, 1.14)	1.02 (0.91, 1.13)	1.00 (0.87, 1.15)	1.07 (0.97, 1.19)	0.131
Additionally adjusted tea drinking§	1.00	1.03 (0.91, 1.15)	1.03 (0.93, 1.15)	1.02 (0.88, 1.17)	1.08 (0.97, 1.19)	0.146

*All sensitivity analyses were conducted based on model 2. The regions mainly consuming salted vegetables included Qingdao, Harbin, Suzhou, and Zhejiang.

†Dietary supplements included fish oil/cod liver oil, vitamins, calcium/iron/zinc, ginseng, and other herbal products (regular intake or not).

‡Other food groups included rice, wheat, other staple, poultry, fish/sea food, eggs, soybean, and dairy products (never/rarely, monthly, 1-3 days/week, 4-6 days/week, or daily).

§The status of tea drinking was grouped according to the frequency and temperature of tea, including less than weekly, weekly, daily and warm, daily and hot, or daily and burning hot.

‖This endpoint is the first incident gastrointestinal tract cancer (which could be either oesophageal, stomach, or colorectal cancer).

Table S9. Sensitivity analyses for the association between preserved vegetable consumption and gastrointestinal tract cancers in the regions mainly consuming sour pickled vegetables (n=105,372)*

Types of cancer	Frequency of preserved vegetable consumption					*P* for trend
	Never	Monthly	1-3 days/week	4-6 days/week	Daily	
**Oesophageal cancer**						
Model 2	1.00	1.02 (0.73, 1.42)	1.07 (0.80, 1.42)	1.14 (0.82, 1.58)	1.35 (1.02, 1.80)	0.013
Excluding cases in the first 2 years of follow-up	1.00	1.16 (0.81, 1.66)	1.10 (0.80, 1.50)	1.14 (0.79, 1.64)	1.44 (1.05, 1.96)	<0.001
Additionally adjusted daily energy intake	1.00	1.02 (0.73, 1.42)	1.07 (0.80, 1.42)	1.14 (0.82, 1.58)	1.36 (1.02, 1.81)	0.012
Additionally adjusted intake of dietary supplements†	1.00	1.02 (0.73, 1.42)	1.07 (0.80, 1.42)	1.14 (0.82, 1.58)	1.35 (1.02, 1.80)	0.013
Additionally adjusted the years of owning a refrigerator	1.00	1.02 (0.73, 1.42)	1.07 (0.80, 1.42)	1.14 (0.82, 1.58)	1.35 (1.02, 1.80)	0.013
Additionally adjusted other food groups consumption‡	1.00	1.03 (0.73, 1.43)	1.07 (0.80, 1.42)	1.15 (0.82, 1.60)	1.34 (1.00, 1.78)	0.018
Additionally adjusted tea drinking§	1.00	1.03 (0.74, 1.43)	1.06 (0.80, 1.41)	1.13 (0.81, 1.57)	1.34 (1.01, 1.78)	0.018
**Stomach cancer**						
Model 2	1.00	0.80 (0.62, 1.03)	0.86 (0.69, 1.07)	0.86 (0.66, 1.10)	0.91 (0.71, 1.16)	0.771
Excluding cases in the first 2 years of follow-up	1.00	0.75 (0.57, 0.99)	0.79 (0.62, 1.01)	0.74 (0.56, 0.98)	0.91 (0.70, 1.18)	0.575
Additionally adjusted daily energy intake	1.00	0.80 (0.62, 1.03)	0.86 (0.69, 1.07)	0.85 (0.66, 1.10)	0.91 (0.71, 1.16)	0.784
Additionally adjusted intake of dietary supplements†	1.00	0.80 (0.62, 1.03)	0.86 (0.69, 1.07)	0.85 (0.66, 1.10)	0.91 (0.71, 1.16)	0.766
Additionally adjusted the years of owning a refrigerator	1.00	0.80 (0.62, 1.04)	0.86 (0.69, 1.07)	0.86 (0.66, 1.10)	0.91 (0.71, 1.16)	0.771
Additionally adjusted other food groups consumption‡	1.00	0.81 (0.63, 1.05)	0.87 (0.70, 1.09)	0.85 (0.66, 1.09)	0.90 (0.71, 1.16)	0.671
Additionally adjusted tea drinking§	1.00	0.81 (0.62, 1.04)	0.86 (0.69, 1.08)	0.85 (0.66, 1.10)	0.91 (0.71, 1.17)	0.763
**Colorectal cancer**						
Model 2	1.00	1.53 (1.09, 2.14)	1.45 (1.07, 1.97)	1.35 (0.95, 1.94)	1.37 (0.99, 1.89)	0.292
Excluding cases in the first 2 years of follow-up	1.00	1.60 (1.11, 2.29)	1.54 (1.11, 2.14)	1.46 (1.00, 2.14)	1.45 (1.03, 2.06)	0.260
Additionally adjusted daily energy intake	1.00	1.53 (1.09, 2.14)	1.44 (1.06, 1.96)	1.35 (0.95, 1.94)	1.36 (0.98, 1.88)	0.322
Additionally adjusted intake of dietary supplements†	1.00	1.53 (1.09, 2.14)	1.45 (1.07, 1.97)	1.35 (0.95, 1.94)	1.37 (0.99, 1.90)	0.292
Additionally adjusted the years of owning a refrigerator	1.00	1.53 (1.09, 2.14)	1.45 (1.07, 1.97)	1.35 (0.95, 1.94)	1.37 (0.99, 1.89)	0.291
Additionally adjusted other food groups consumption‡	1.00	1.50 (1.07, 2.11)	1.44 (1.06, 1.96)	1.37 (0.95, 1.96)	1.39 (1.00, 1.92)	0.224
Additionally adjusted tea drinking§	1.00	1.53 (1.09, 2.15)	1.45 (1.07, 1.96)	1.35 (0.95, 1.94)	1.36 (0.99, 1.88)	0.313

Types of cancer	Frequency of preserved vegetable consumption					*P* for trend
	Never	Monthly	1-3 days/week	4-6 days/week	Daily	
**Gastrointestinal tract cancers‖**						
Model 2	1.00	1.04 (0.87, 1.24)	1.07 (0.92, 1.25)	1.07 (0.90, 1.28)	1.16 (0.98, 1.36)	0.072
Excluding cases in the first 2 years of follow-up	1.00	1.07 (0.88, 1.29)	1.08 (0.91, 1.27)	1.04 (0.85, 1.26)	1.20 (1.00, 1.43)	0.075
Additionally adjusted daily energy intake	1.00	1.04 (0.87, 1.24)	1.07 (0.92, 1.25)	1.07 (0.90, 1.28)	1.16 (0.98, 1.36)	0.072
Additionally adjusted intake of dietary supplements†	1.00	1.04 (0.87, 1.24)	1.07 (0.92, 1.25)	1.07 (0.90, 1.28)	1.15 (0.98, 1.36)	0.074
Additionally adjusted the years of owning a refrigerator	1.00	1.04 (0.87, 1.24)	1.07 (0.92, 1.25)	1.07 (0.90, 1.28)	1.16 (0.98, 1.36)	0.073
Additionally adjusted other food groups consumption‡	1.00	1.04 (0.87, 1.24)	1.08 (0.92, 1.26)	1.08 (0.90, 1.29)	1.15 (0.98, 1.36)	0.083
Additionally adjusted tea drinking§	1.00	1.04 (0.87, 1.24)	1.07 (0.92, 1.25)	1.07 (0.90, 1.28)	1.15 (0.98, 1.36)	0.083

*All sensitivity analyses were conducted based on model 2. The regions mainly consuming sour pickled vegetables included Sichuan and Gansu.

†Dietary supplements included fish oil/cod liver oil, vitamins, calcium/iron/zinc, ginseng, and other herbal products (regular intake or not).

‡Other food groups included rice, wheat, other staple, poultry, fish/sea food, eggs, soybean, and dairy products (never/rarely, monthly, 1-3 days/week, 4-6 days/week, or daily).

§The status of tea drinking was grouped according to the frequency and temperature of tea, including less than weekly, weekly, daily and warm, daily and hot, or daily and burning hot.

‖This endpoint is the first incident gastrointestinal tract cancer (which could be either oesophageal, stomach, or colorectal cancer).

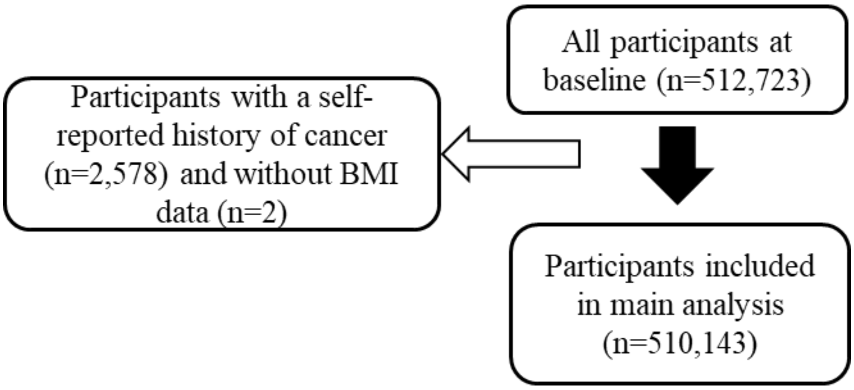

Figure S1. Participant flow chart

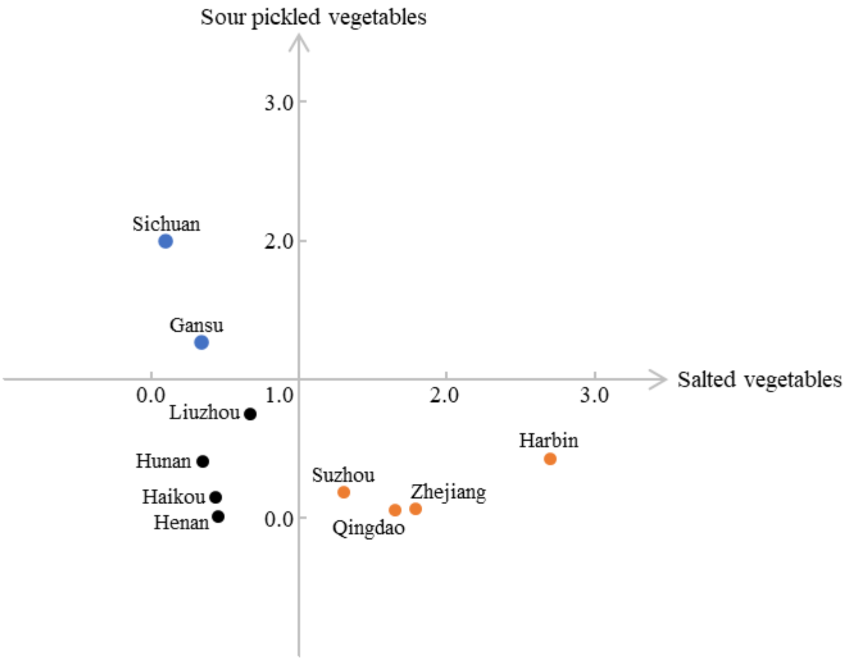

Figure S1. The average intake level of salted and sour picked vegetables in the second resurvey (days/week)

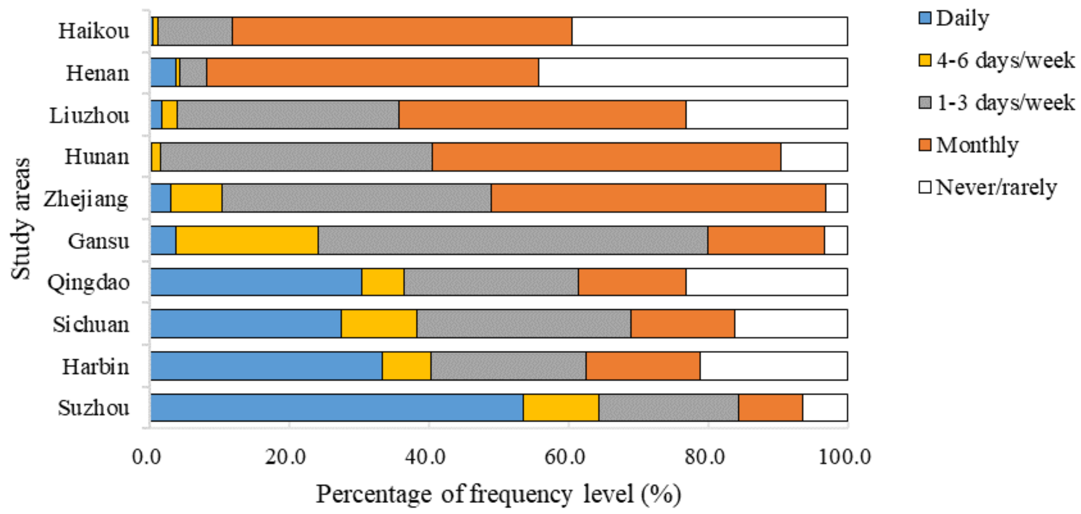

Figure S3. Frequency of preserved vegetable consumption in CKB study areas

Reference

1 Kakkoura MG, Du H, Guo Y, Yu C, Yang L, Pei P, et al. Dairy consumption and risks of total and site-specific cancers in Chinese adults: an 11-year prospective study of 0.5 million people. BMC Med. 2022;20(1):134.

2 Wei Y, Lv J, Guo Y, Bian Z, Gao M, Du H, et al. Soy intake and breast cancer risk: a prospective study of 300,000 Chinese women and a dose – response meta-analysis. Eur J Epidemiol. 2020;35(6):567-78.
